# Displacement and Strain Measurement up to 1000 °C Using a Hollow Coaxial Cable Fabry-Perot Resonator

**DOI:** 10.3390/s18051304

**Published:** 2018-04-24

**Authors:** Chen Zhu, Yizheng Chen, Yiyang Zhuang, Jie Huang

**Affiliations:** Department of Electrical and Computer Engineering, Missouri University of Science and Technology, Rolla, MO 65409, USA; cznwq@mst.edu (C.Z.); ycb28@mst.edu (Y.C.); yz8r4@mst.edu (Y.Z.)

**Keywords:** high temperature, displacement, strain, hollow coaxial cable, Fabry-Perot resonator

## Abstract

We present a hollow coaxial cable Fabry-Perot resonator for displacement and strain measurement up to 1000 °C. By employing a novel homemade hollow coaxial cable made of stainless steel as a sensing platform, the high-temperature tolerance of the sensor is dramatically improved. A Fabry-Perot resonator is implemented on this hollow coaxial cable by introducing two highly-reflective reflectors along the cable. Based on a nested structure design, the external displacement and strain can be directly correlated to the cavity length of the resonator. By tracking the shift of the amplitude reflection spectrum of the microwave resonator, the applied displacement and strain can be determined. The displacement measurement experiment showed that the sensor could function properly up to 1000 °C. The sensor was also employed to measure the thermal strain of a steel plate during the heating process. The stability of the novel sensor was also investigated. The developed sensing platform and sensing configurations are robust, cost-effective, easy to manufacture, and can be flexibly designed for many other measurement applications in harsh high-temperature environments.

## 1. Introduction

High-temperature displacement and strain measurement are of high importance in various applications such as monitoring of jet engines or power turbines, characterizing the mechanical and thermophysical properties of the materials, movement control and position monitoring in harsh high-temperature environments, steel-making industries, advanced energy systems, and space exploration [1]. Linear variable differential transformers (LVDTs) are so far the most widely used displacement and strain sensors, from laboratory experiments to industrial applications. A typical LVDT consists of three solenoidal coils (i.e., two secondary coils and a primary coil) and a center cylindrical ferromagnetic core. As the core moves, the coupling ratios of the magnetic flux produced by the primary coil to the two secondary coils become different, which induces different voltages in the two secondary coils [2]. Due to the working mechanism, the highest temperature tolerance of LVDTs reported is 650 °C [3]. Another widely used method for high-temperature displacement and strain measurement is non-contact imaging, such as digital image correlation (DIC), which has been demonstrated for full-field deformation measurements at a temperature up to 1200 °C [4]. However, in high-temperature environments, blackbody radiation from the object of interest will inevitably affect image quality. Meanwhile, some environmental factors such as humidity and vibration have a detrimental influence on the performance of this imaging instrument.

In recent years, optical fiber sensors have been widely demonstrated for strain and displacement measurement in various applications, showing great advantages such as immunity to electromagnetic interference, high sensitivity, high resolution, remote operation, and distributed sensing capabilities [5,6]. A variety of techniques have been developed for implementing strain or displacement sensors, such as fiber Bragg gratings (FBG) [7], Mach-Zehnder interferometers [8], Michelson interferometers [9], and Fabry-Perot interferometers (FPIs) [10,11]. Most optical fiber sensors are engineered on silica glass optical fibers [12], which are primarily developed for long-haul telecommunication. The fiber core of the widely used single mode fibers (SMFs) is typically made of fused silica with dopants (e.g., germanium oxide) [13]. When it comes to high-temperature applications (e.g., 800 °C), the long-term stability and durability of the SMF-based sensors become a concern due to the softening of silica glass and the migration/diffusion of the dopants inside the optical fiber core. For instance, a signal drift in the output spectrum of an FBG occurs due to the diffusion of the dopants especially for temperatures beyond 650 °C [14]. To address the long-term stability and durability issues faced by SMF-based sensors under high-temperature environments, sapphire optical fiber has been proposed for sensing applications in high-temperature environments [15,16]. However, sapphire optical fibers support thousands of optical modes with different traveling speeds due to their large core diameters or uncladded structures. Thus, it is very difficult to migrate the sensing ideas that have been used on SMFs to the sapphire fibers. On the other hand, most of the optical fiber displacement sensors work in a bending structure, which gives rise to a concern regarding long-term stability [17,18]. In addition, optical fibers are fragile and can be easily broken down when subjected to a large tensile strain (e.g., above 4000 µε) or a shear force [19]. Consequently, packaging, installing, and maintaining optical fiber sensors, especially in harsh high-temperature environments, are always a challenge. Despite the great efforts that have been made on the development of optical fiber sensors, these drawbacks still hinder their potential applications for displacement and strain measurement in high-temperature environments.

One solution to the problem is to find another type of cable as the signal transmission medium. Our search led to the coaxial cable. Coaxial cable and optical fiber are the two basic forms of transmission lines that have been widely used in telecommunications for transmitting signals over a long distance. Governed by the same electromagnetic (EM) theory, the two types of transmission lines share the common fundamental physics. Typically, a coaxial cable is formed by an inner conductor, an outer conductor, and a dielectric layer sandwiched in-between the two conductors. In recent years, some of the fiber optic sensing technologies have been adopted onto coaxial cables [20,21,22,23,24,25,26,27,28,29]. This is because coaxial cables are much more robust than optical fibers regarding mechanical strength. Thus, some of the challenging issues (e.g., fragility, bend sensitivity, and difficulty in employing) faced by optical fiber sensors are no longer a concern for coaxial cable sensors. For example, coaxial cable Bragg gratings (CCBGs) [20,21] and coaxial cable Fabry-Perot interferometers (CCFPIs) [22,23] have already been demonstrated effective for large strain measurements. Recently, we proposed a coaxial cable Fabry-Perot resonator (CCFPR) for temperature measurement in the range of 35 to 80 °C [26]. The working principle of these coaxial cable sensors is that the change in strain or temperature will induce an elongation or compression of coaxial cable sensors. Due to the dielectric layer (e.g., polyethylene) inside the traditional coaxial cable and the outer plastic jacket, these sensors can only survive up to 200 °C. An interesting question is whether the outer jacket and insulating layer of the coaxial cable sensors can be removed. This is not only interesting from a physics perspective but also motivated by the application potential that such a coaxial cable sensor (without a plastic jacket and an insulating polymer layer) could provide a promising solution for high-temperature sensing applications. In addition, such a coaxial cable has great potential to be modified for many other high-temperature sensing applications such as pressure/acoustic sensor, liquid level meter (molten iron level meter in steel-making industry), accelerometer, tilt meter, and embeddable strain gauge, etc.

Motivated by the demand of high-temperature applications, in this paper, we report on a novel homemade hollow coaxial cable Fabry-Perot resonator (HCC-FPR) for high-temperature strain and displacement measurements up to 1000 °C. The HCC consists of a stainless steel tube as an outer conductor and a stainless steel round bar as an inner conductor with air in between. Inspired by the optical fiber FPR sensors [30], a microwave FPR structure was fabricated on an HCC for sensing applications. In the FPR design, a cylindrical stainless steel post is used to short the inner conductor and outer conductor, serving as the first highly reflective reflector (reflectivity: ~97% at 1 GHz) due to large impedance mismatch; a stainless steel sheet is welded on the end of the HCC, which also shorts the inner conductor and outer conductor, thus serving as the second reflector (reflectivity: ~100% at 1 GHz). Based on a nested structure design, the second reflector can relatively slide with respect to the first reflector. When the FPR is subjected to a displacement or strain, the cavity length, defined as the distance between the two reflectors, will change due to the movement of the second reflector. By tracking the shift in the amplitude reflection spectrum, the change in the cavity length of the FPR can be calculated, making the HCC-FPR act as a sensor device. In the demonstration experiments, the novel sensor was employed to measure displacements under various temperatures (125 to 1000 °C). Based on the same structure design and working principle, a strain sensor was developed, and the sensor was verified to quantify the thermal strain of a steel plate up to 900 °C. The experimental results showed that such sensors can achieve high-temperature displacement and strain measurements with good sensitivity and stability.

## 2. Sensor Design and Measurement Principle

### 2.1. The HCC Design

**Figure 1:**
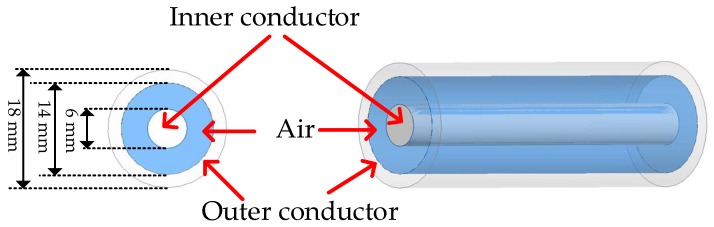
A schematic drawing of the hollow coaxial cable (HCC). The HCC consists of an inner conductor, an outer conductor, and air serving as a dielectric layer.

**Figure 2:**
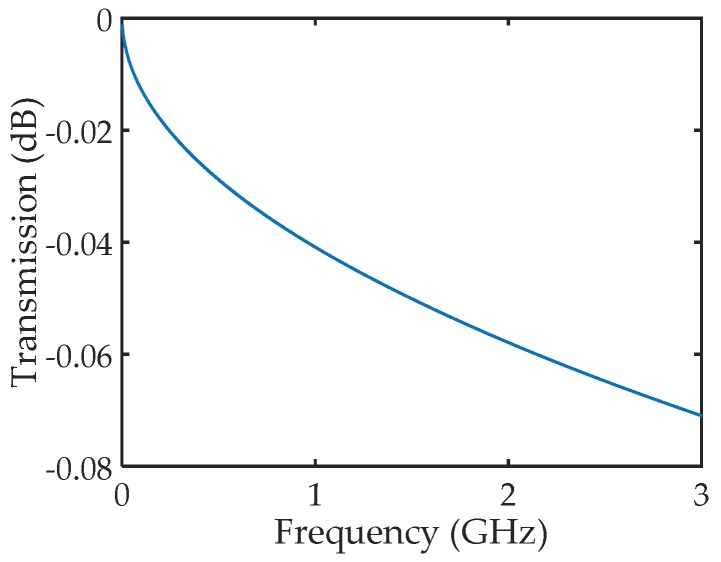
The transmission loss of an 11.8-cm-long HCC as a function of frequency simulated by ANSYS HFSS.

A schematic drawing of the homemade HCC is shown in Figure 1. The HCC has a solid inner conductor and an outer conductor. The dielectric layer in-between the two conductors is air. Spacers will be added to support this hollow structure (i.e., internal reflectors and connectors). The diameter of the inner conductor is designed to be 6 mm, and the outer diameter of the dielectric layer (air in between) is designed to be 14 mm. This structure design achieves an impedance of 50.8 Ω for the HCC [31]. Meanwhile, based on the design, the HCC is also capable of being connected to a commercial coaxial cable (e.g., RG-58/U) via an SMA to Type N adapter. The outer diameter of the outer conductor is designed to be 18 mm to make the HCC robust. It should be noted that the HCC is user-configurable, which could be designed to have different sizes, such as 3/7 mm or 2/4.6 mm (diameter of the inner conductor/outer diameter of the air dielectric layer), for different applications. The transmission loss of the novel HCC was numerically simulated using ANSYS HFSS. Figure 2 plots the loss of an 11.8-cm-long HCC as a function of frequency. As can be seen, the transmission loss of the novel HCC is estimated to be less than 1 dB/m for the range of 0–3 GHz, meaning that the HCC has the potential to be a new sensing platform for long-distance distributed sensing applications. Type 310S Stainless Steel is employed to fabricate the HCC for elevated-temperature applications. For extremely high-temperature environments (e.g., 1600 °C), other metals with higher melting points (e.g., niobium, molybdenum, tantalum, and tungsten) can be employed to fabricate the HCC. 

### 2.2. The HCC-FPR Design

**Figure 3:**
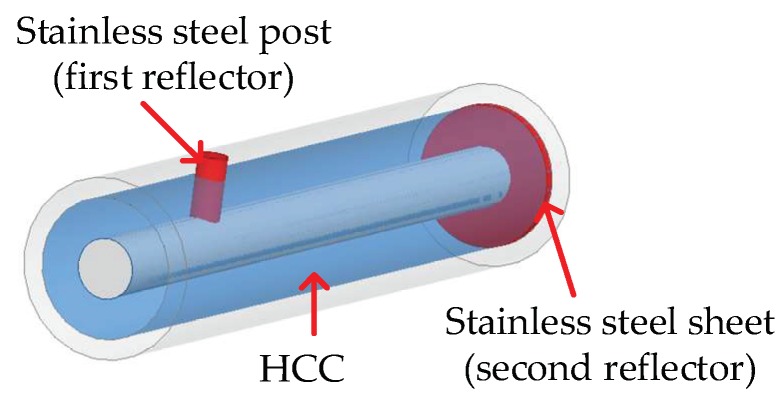
A schematic drawing of the developed hollow coaxial cable Fabry-Perot resonator (HCC-FPR).

In a transmission line, there are several ways to introduce a large impedance mismatch to form a reflector with high reflectivity (e.g., >50%) to the EM wave propagating inside the waveguide, including varying the radius ratio of the inner/outer conductors, varying the dielectric materials, and shorting the inner/outer conductors. In the HCC-FPR design, a cylindrical stainless steel post, which shorts the inner and outer conductors, serves as the first highly reflective reflector; a stainless steel sheet, which also shorts the two conductors, serves as the second highly reflective reflector. The distance between the two reflectors can range from several centimeters to tens of centimeters. A schematic drawing of the developed HCC-FPR is presented in Figure 3. It should be noted that the two reflectors can be of different shapes (e.g., cubical and conical) [26].

When a microwave signal is launched into the HCC-FPR, most of the EM wave is reflected by the first reflector, and the rest of the EM wave travels through the cavity and reaches the second reflector. Again, most of the signal gets reflected by the second reflector. Multiple reflections and multiple wave interference occur inside the resonator formed by the two reflectors. The phase delay (*δ*) between two reflected waves is given by

(1) $$\delta =\frac{4\pi d}{\lambda }=\frac{4\pi fd\sqrt{\varepsilon_{r}}}{c}$$

where *λ* and *f* are the wavelength and frequency of the EM wave, respectively; *d* is the resonance cavity length; *ε_r_* is the effective dielectric constant of air inside the cavity; *c* is the speed of light in vacuum. For infinite reflections in the resonant cavity, the reflection coefficient of the FPR can be expressed as

(2) $$r=\frac{r_{1}-r_{2}v^{2}\exp(j\delta )}{1-r_{1}r_{2}v^{2}\exp(j\delta )}$$

where *r*_1_ and *r*_2_ are the reflection coefficients of the first reflector and the second reflector, respectively; *v* is the loss factor of the cavity, which represents the fraction of the incident wave amplitude that is transmitted by the cavity. The amplitude (*A*) of the reflected electric field by the FPR can be calculated by

(3) $$A=\sqrt{\frac{r_{1}{}^{2}-2r_{1}r_{2}v^{2}\cos\delta +{\left(r_{2}v^{2}\right)}^{2}}{1-2r_{1}r_{2}v^{2}\cos\delta +r_{1}{}^{2}{\left(r_{2}v^{2}\right)}^{2}}}.$$

Meanwhile, multiple resonant dips would be observed at the frequencies where the phase delay (*δ*) is equal to 2*mπ* (*m* is an integer, i.e., 1, 2, 3…). Thus, the resonant frequency (*f_res_*) and resonant wavelength can be determined by

(4) $$\begin{array}{l} f_{res}=m\frac{c}{2d\sqrt{\varepsilon_{r}}}\\ \lambda_{res}=\frac{2}{m}d\sqrt{\varepsilon_{r}} \end{array}.$$

**Figure 4:**
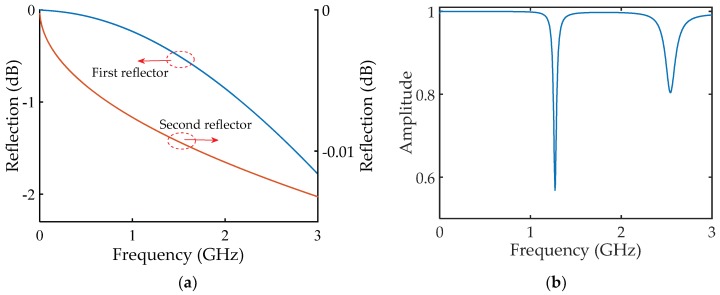
Calculated results of the HCC-FPR. (**a**) Simulated reflection coefficients of the two reflectors; (**b**) Calculated amplitude reflection spectrum of the HCC-FPR.

In order to numerically calculate the amplitude reflection spectrum *A* of the resonator using Equation (3), the reflection coefficients of the two reflectors were simulated using ANSYS HFSS. The simulation results are plotted in Figure 4a. As can be seen, both of the two reflectors have very high reflectivities in the observation bandwidth (0–3 GHz), and both of the reflectivities decrease as frequency increases. Zero dB shown in Figure 4a represents 100% reflectivity in amplitude with respect to the incident EM waves. The reflectivity of the second reflector (i.e., the stainless steel sheet) is higher than that of the first reflector (i.e., the cylindrical stainless steel post). They both drop at higher frequency regions. Setting the cavity length of the resonator to be 11.8 cm, and substituting the simulated reflection coefficients of the two reflectors and loss factor of the HCC (shown in Figure 2), the calculated amplitude reflection spectrum of the resonator in the range of 0–3 GHz is shown in Figure 4b. Two resonant dips can be clearly identified, including the fundamental resonant frequency at 1.271 GHz and the second harmonic at 2.542 GHz. The quality factors (Q-factors) of the two valleys are determined to be 36 and 25, respectively, indicating a resonant device (i.e., FPR) is developed. It should be noted that, compared with an optical fiber FPR, the stringent requirements such as high manufacturing accuracy (e.g., the surface roughness is typically 1/20 of the operation wavelength) and precise alignment, can be largely relieved in fabricating such an HCC-FPR. Integrated on a coaxial cable sensing platform, the HCC-FPR can be a good candidate for remote sensing applications in harsh high-temperature environments.

### 2.3. HCC-FPR Displacement Sensor Design

**Figure 5:**
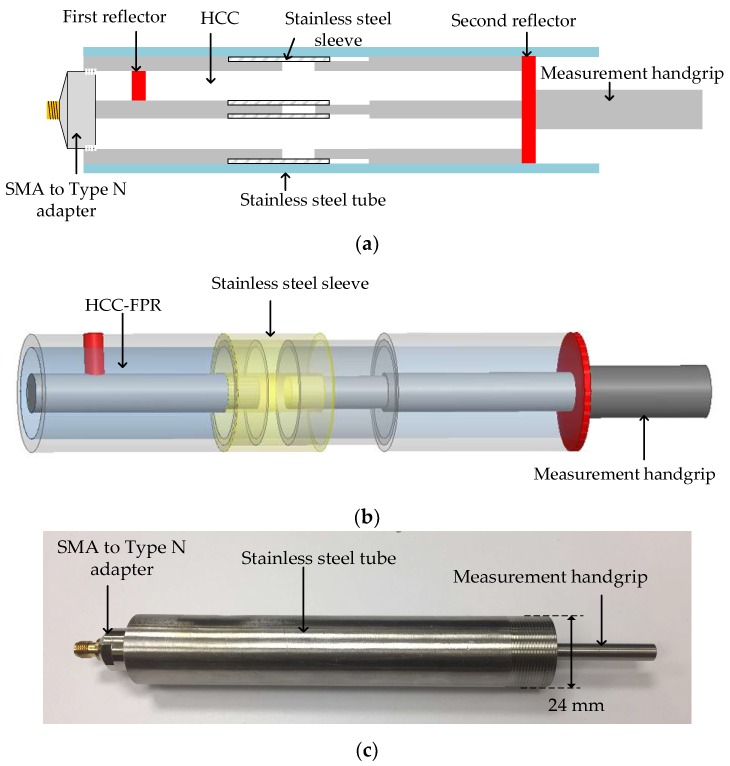
The proposed HCC-FPR-based displacement sensor. (**a**) A schematic drawing of the displacement sensor; (**b**) Three-dimensional rendering of the sensor. The outer stainless steel tube is not shown in the three-dimensional drawing; (**c**) A photograph of the prototype HCC-FPR-based displacement sensor. Due to the sleeve design, the right HCC part, including the second reflector will move with respect to the left HCC part when a displacement is applied to the measurement handgrip.

As mentioned above, the HCC-FPR is a resonant device with several resonant frequencies in the amplitude reflection spectrum, and the resonant frequencies are a function of the cavity length of the resonator. In the HCC-FPR displacement sensor design, the idea is to correlate the external displacement to the cavity length of the HCC-FPR. One way is to make the steel sheet (shown in Figure 3) movable. However, we found that movable steel sheet (i.e., the second reflector) had a contact issue with the inner/outer conductors such that the displacement measurement resolution was limited to the thickness of the steel sheet. In order to have a stable and solid physical contact between the steel sheet and the inner/outer conductors, a novel design for the high-temperature HCC-FPR displacement sensor is proposed and shown in Figure 5a–c. Figure 5a shows a schematic drawing of the novel HCC-FPR-based displacement sensor; Figure 5b shows a three-dimensional rendering of the sensor; Figure 5c shows a photograph of the prototype sensor. There are two separate HCC parts. The first reflector is welded to the left HCC part. The second reflector is welded to the right HCC part, which is movable due to the stainless steel sleeve design and is also permanently connected to a measurement handgrip made of stainless steel. When the measurement handgrip is subjected to an external displacement, the right HCC part and the second reflector will also move, leading to a change in the cavity length of the FPR. Thus, the external displacement is directly correlated to the cavity length of the resonator (i.e., the magnitude of the displacement is equal to the magnitude of the cavity length change). The stainless steel tube is designed to regulate the movement direction of the right HCC part and to protect the main body of the HCC-FPR sensor.

According to Equation (4), for the fundamental resonant dip (i.e., *m* = 1), the resonant frequency is not linearly proportional to the cavity length (i.e., in reciprocal proportion), while the resonant wavelength is linearly proportional to the cavity length. Hence, in the demodulation process, we correlate the displacement to the resonant wavelength rather than the resonant frequency. For the fundamental mode, the shift in the resonant wavelength (Δ*λ*) due to the cavity length change (Δ*d*) can be expressed as

(5) $$\Delta \lambda =2\sqrt{\varepsilon_{r}}\Delta d.$$

## 3. Experimental Results and Discussions

**Figure 6:**
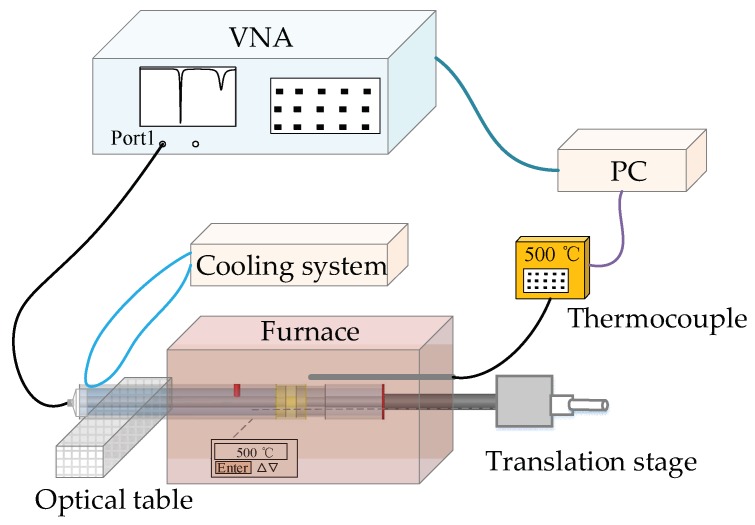
A schematic drawing of the experimental setup.

The experimental setup for high-temperature displacement measurements is schematically presented in Figure 6. A vector network analyzer (VNA, Agilent 8753ES, Santa Clara, CA USA) was used as the signal source and detector. Port 1 of the VNA was connected to the HCC-FPR-based displacement sensor through a flexible coaxial cable (RG-58/U). The flexible cable is connected to the sensor through an SMA to Type N adapter. The VNA was set with an intermediate frequency bandwidth (IFBW) of 300 Hz, 1601 sampling points, and a scanning bandwidth of 3 GHz (i.e., from 100 KHz to 3 GHz). The scattering parameter S_11_ (i.e., amplitude reflection spectrum) was recorded. The HCC-FPR sensor was placed in a furnace (LINDBERG BLUE M, Thermo SCIENTIFIC, Waltham, MA USA), where a thermocouple was also placed to monitor/calibrate the temperature around the HCC-FPR in real time. A personal computer (PC) was connected to the VNA through a GPIB cable to set the acquisition parameters, acquire the data, and process the data. The PC was also connected to the thermocouple to record the temperature information in real time. In the displacement measurement experiment, one end of the HCC-FPR (e.g., the end with the SMA to Type N adapter) was mounted on an optical table, and the other end (i.e., the end with the measurement handgrip) was positioned by a translation stage. Due to the high temperature in the experiment, an HCC-FPR with a long lead-in HCC (40 cm in length) was employed in the experiment. Meanwhile, a home-made cooling system with a syringe pump filled with tap water was used to continuously cool down the lead-in HCC in real time to prevent the flexible coaxial cable from heating. In practical high-temperature applications, a long ceramic or hollow coaxial cable can be used as a lead-in cable for signal transmission, or if possible, a wireless RF interrogation unit can be designed to prevent heat transfer from the sensor to the interrogator.

**Figure 7:**
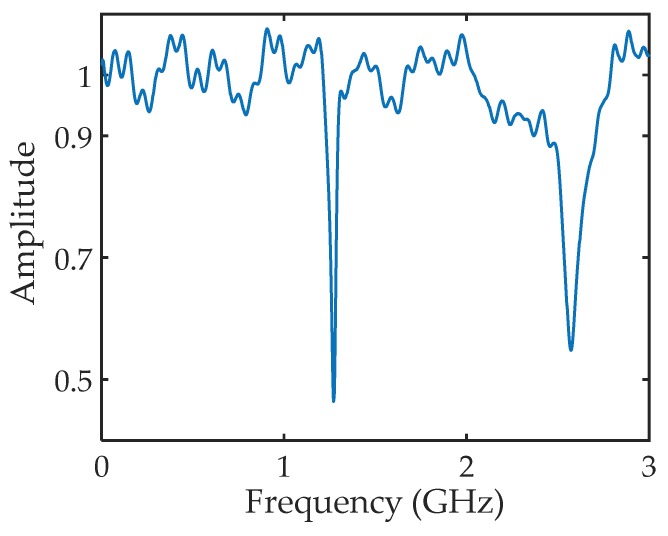
Experimentally obtained amplitude reflection spectrum of the HCC-FPR displacement sensor under a cavity length setting of 11.8 cm.

The recorded amplitude reflection spectrum of the prototype sensor under a cavity length setting of 11.8 cm is plotted in Figure 7. Two resonant dips were obtained, which matches well with the calculated results, as shown in Figure 4b, and the Q-factors of the two dips are calculated to 35 and 23, respectively, indicating that the proposed HCC-FPR concept works. The contrast of the second-order resonant dip in the obtained reflection spectrum is larger than the simulation result (i.e., Figure 4b). The reason is that the loss of the HCC in the experiment is larger than the simulation result (i.e., Figure 2), resulting in a more balanced condition regarding the reflectivities of the two reflectors compared with the theoretical calculations. The small ripples in the reflection spectrum are due to the multiple reflections incurred from Port 1 of the VNA and the SMA to Type N adapter.

### 3.1. High-Temperature Displacement Measurement

**Figure 8:**
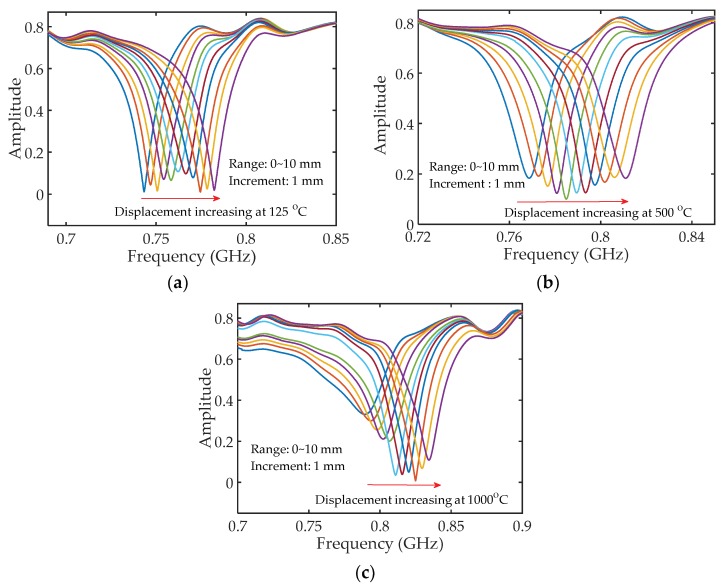
The shift in the resonant spectra of the HCC-FPR displacement sensor as the displacement increased from 0 to 10 mm with a step of 1 mm at three different temperature settings. (**a**) 125 °C; (**b**) 500 °C; (**c**) 1000 °C.

**Figure 9:**
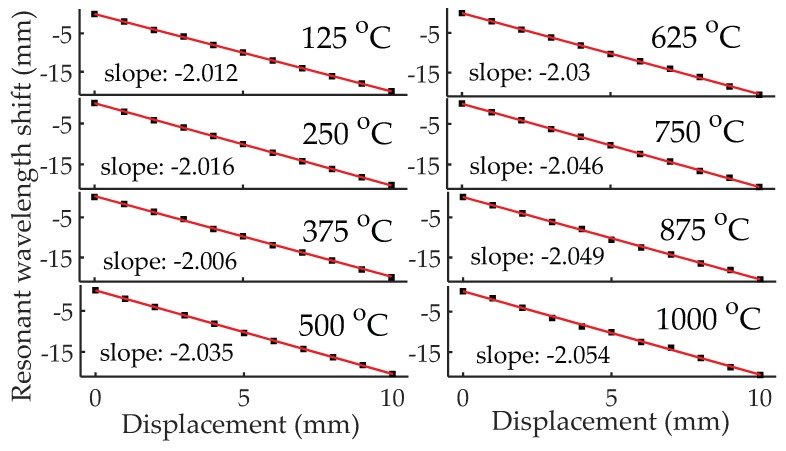
The shift in the resonant wavelength as a function of displacement over a displacement range of 10 mm with a step of 1 mm at eight different temperature settings (125–1000 °C with a step of 125 °C). Linear curve fitting was applied to the test data, and the slopes are indicated in the figure.

In the high-temperature displacement measurement experiment, the displacement was applied to the measurement handgrip using a translation stage with a resolution of 0.01 mm. At each temperature setting (i.e., 125 to 1000 °C with a step of 125 °C), the displacement was increased from 0 to 10 mm with a step of 1 mm. After finishing the displacement measurements at each temperature setting, the measurement handgrip of the sensor was pulled back. Figure 8 shows the recorded reflection spectra of the sensor for displacement measurements at three different temperature settings: 125, 500, and 1000 °C. As can be seen from the three figures, the resonant frequency shifted to higher frequency region as the displacement increased, which matches well with our expectation. When the displacement increases, the cavity length decreases (Δ*d* = −Δ*d*’, Δ*d*’ is the applied displacement), leading to a higher resonant frequency according to Equation (4). The Q-factors of the resonant dips decreased as the temperature increased from 125 to 1000 °C. In addition, the loss of the HCC-FPR at 1000 °C was larger than that of the HCC at 125 °C, which is a reason for the decrease in the Q-factors. We believe that the increased loss of the HCC in elevated temperature is due to the oxidation of the stainless steel and the increased kinetic energy of the metal atoms (i.e., increased resistance). On the other hand, the Q-factor of the resonant dips in Figure 8c (i.e., displacement measurement at 1000 °C) varied, which was partially caused by the high loss in high-temperature setting. The larger the displacement, the smaller the cavity length is, so the round-trip loss between the two reflectors becomes smaller, leading to a larger Q-factor of the resonant dip as indicated in Figure 8c. Another reason could be that, in high-temperature environments, the physical contact between the left HCC-part and right HCC-part is not as good as that at room temperature due to thermal expansion, resulting in an unexpected loss. Figure 9 shows the shift in the resonant wavelength (converted from the resonant frequency) as a function of applied displacement at eight different temperature settings. Linear curve fitting was applied to each data set at different temperature settings. The sensitivities (i.e., the fitted slope as shown in the figure), defined as the ratio of the resonant wavelength shift to the applied displacement, were determined to be −2.012 mm/mm, −2.016 mm/mm, −2.006 mm/mm, −2.035 mm/mm, −2.030 mm/mm, −2.046 mm/mm, −2.049 mm/mm, and −2.054 mm/mm for eight different temperature settings, demonstrating that the developed HCC-FPR-based sensor can be used as a displacement sensor at high-temperature environments up to 1000 °C. The differences between the measured sensitivities and the theoretical sensitivity ($-2\sqrt{\varepsilon_{r}}$, −2.000 mm/mm if $\varepsilon_{r}=1$) are mainly because the relative permittivity of air is different at different temperatures.

### 3.2. High-Temperature Strain Measurement

**Figure 10:**
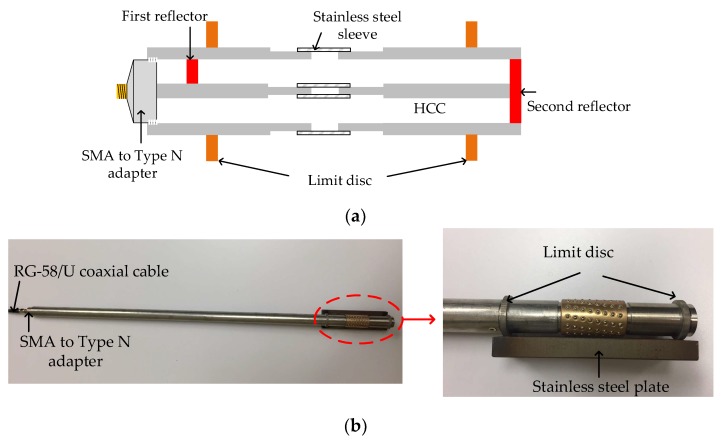
The proposed HCC-FPR-based strain sensor. (**a**) A schematic drawing of the strain sensor. Two limit discs are welded on the HCC-FPR; (**b**) A photograph of the prototype HCC-FPR-based sensor for thermal strain measurement. The distance between the two limit discs is 11.5 cm.

Based on the developed HCC-FPR displacement sensing configuration, a high-temperature strain sensor was also designed, fabricated, and tested. A schematic drawing of the HCC-FPR strain sensor is shown in Figure 10a. Two limit discs are welded on the HCC-FPR. When the two limit discs are subjected to a compression or tension, due to the nested structure design, both the left HCC part (including the first reflector) and the right HCC part (including the second reflector) will move, leading to a change in the cavity length of the FPR. It should be noted that, because of the nested structure design, the slide between the left HCC part and the right HCC part has low friction, which means that the sensor can be employed for strain measurement of materials with low Young’s modulus. To demonstrate the sensor’s capability for high-temperature strain measurement, the sensor was used to measure the thermal strain of a steel plate during the heating process. Figure 10b shows a photograph of the thermal strain test sensor, in which a stainless steel plate is welded on the two limit discs. The distance between the two limit discs is 11.5 cm. The figure on the right in Figure 10b is an enlarged figure of the marked area in the figure on the left, which clearly shows the HCC-FPR sensor part. Similarly, due to the high temperature, an HCC-FPR with a long lead-in HCC was used in the experiment. We expect that the thermal expansion of the stainless steel plate can be precisely captured by the “frictionless” HCC-FPR strain sensor.

Importantly, after the stainless steel plate is welded on the strain sensor at the two limit discs, due to the two welding points, only the thermal expansion of the stainless steel plate will contribute to the cavity length change of the resonator, while the thermal expansion of the sensor itself will not influence the cavity length due to the nested structure design (i.e., the thermal expansion of the sensor will be compensated by the space in the stainless steel sleeve). The thermal strain (*ε*) of the part of the steel plate between the two limit discs can be expressed as

(6) $$\varepsilon =\frac{\Delta L}{L}=\alpha_{CTE}\Delta T$$

where *L* and Δ*L* are the initial length and length change of the stainless steel plate (the part between the two limit discs) due to temperature change; $\alpha_{CTE}$ is the coefficient of thermal expansion (CTE) of the stainless steel plate; Δ*T* is the change in temperature. Due to the judicious mechanical design, the length change (Δ*L*) of the stainless steel plate is effectively transferred to the cavity length change of the FPR, leading to a resonant wavelength shift (Δ*λ*) in the amplitude reflection spectrum, and the relationship is given by Equation (5). Thus, the relationship between the thermal strain and the resonant wavelength shift can be expressed as

(7) $$\Delta \lambda =2\sqrt{\varepsilon_{r}}\varepsilon L.$$

According to Equations (5)–(7), the CTE of the stainless steel plate can be determined, meaning that the sensor can be employed to characterize the CTEs of different materials at various temperatures.

**Figure 11:**
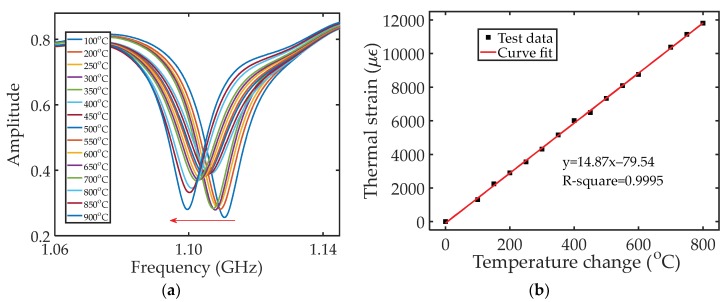
(**a**) Shift in the reflection spectra of the strain sensor under different temperature settings; (**b**) The measured strain as a function of temperature change. Curve fit was applied to the test data.

In the thermal strain experiment, the strain sensor was placed in a furnace. The temperature in the furnace was increased from 100 to 900 °C. Figure 11a plots the recorded amplitude reflection spectra of the strain sensor under different temperature settings. As can be observed, as temperature increased, and the spectrum shifted to a lower frequency region, which is expected based on Equation (4). As temperature increases, the thermal strain of the stainless steel plate increases, leading to an increase in the cavity length of the FPR. The measured relationship between the thermal strain and temperature change is plotted in Figure 11b. Curve fitting was applied to the test data, and a linear relationship was revealed, which matches well with Equation (6). The slope, which is the measured CTE of the stainless steel plate, was determined to be 14.87 × 10^−6^/°C, which matches well with the plate material (stainless steel austenitic 310).

### 3.3. Stability Test

**Figure 12:**
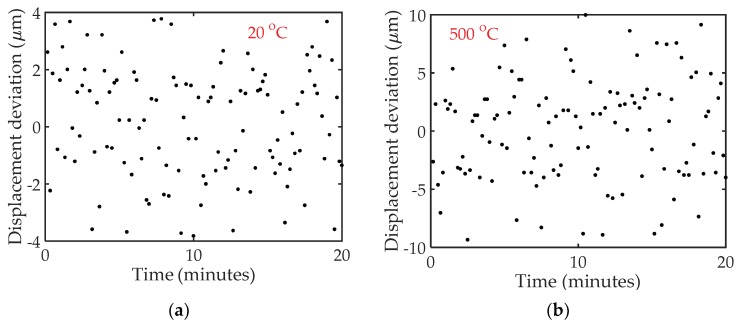
Stability test results at two different temperature settings. (**a**) Measured displacement deviation at room temperature; (**b**) Measured displacement deviation at 500 °C.

The stability of the HCC-FPR-based high-temperature displacement/strain sensor was also investigated. The amplitude reflection spectrum of the sensor for a fixed cavity length was continuously recorded every 10 s for 20 min at room temperature and 500 °C temperature settings. The measured deviations of the displacement as a function of time are plotted in Figure 12. The standard deviations ($\sqrt{\sum_{i=1}^{N}{\left(x_{i}-\bar{x}\right)}^{2}/N-1}$) of the displacement at room temperature and 500 °C temperature settings are determined to be around 2 and 5.5 µm, respectively, indicating good stability of the sensor at different temperature environments. Due to the decrease in Q-factor of the signal and the drift of the furnace at high temperatures, the demodulated displacement deviation increases at high temperature.

**Figure 13:**
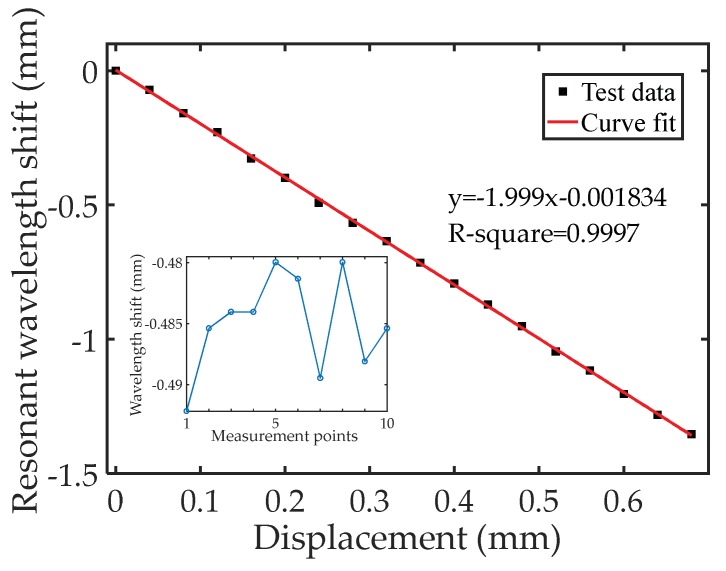
The measured response of the sensor to a displacement range of 0–0.680 mm. The inset shows the measured wavelength shift at a displacement setting of 0.240 mm.

To further demonstrate the measurement accuracy and stability of the proposed displacement sensor, a small range displacement 0–0.680 mm with a step of 0.040 mm was applied to the sensor through a translation stage at room temperature. Figure 13 plots the fundamental resonant wavelength shift as a function of the applied displacement. A linear curve fitting with R-square of 0.9997 indicates a linear relationship between the shift in resonant wavelength and the applied displacement. The slope was found to be −1.999 mm/mm (shift in resonant wavelength/displacement), which agrees well with Equation (5). The inset shows the measured wavelength shift at a displacement setting of 0.240 mm. Ten measured points were recorded. The maximum deviation was calculated to be 12.2 µm, corresponding to a displacement deviation of 6.1 µm, revealing good stability of our sensor.

## 4. Conclusions

In summary, novel HCC-FPR-based displacement and strain sensors were demonstrated for sensing applications up to 1000 °C. Using the novel homemade HCC, the high-temperature tolerance of the sensor was greatly improved. Two highly reflective reflectors, i.e., a cylindrical stainless steel post and a stainless steel sheet, were engineered on an HCC to form a microwave FPR. Based on a nested structure design, the two reflectors can slide, and the slide has very low friction and can be considered as a “frictionless” movement. Due to its inherently frictionless nature, the HCC-FPR has a virtually infinite lifecycle when properly used. A measurement handgrip made of stainless steel is permanently connected to the second reflector. When a displacement is applied to the measurement handgrip of the sensor, the second reflector will move, leading to a cavity length change in the FPR. The change in the cavity length can be determined by tracking the shift in resonant wavelength of the FPR. The displacement responses of the sensor were tested from 125 to 1000 °C, showing good sensitivity and stability in high-temperature environments. Based on the same working principle, a high-temperature strain sensor was also developed. The strain sensor was employed to measure the thermal strain of a stainless steel plate during the heating process from 100 to 900 °C, also showing good stability. The developed HCC-FPR-based sensor has a number of advantages, such as robustness, great high-temperature tolerance, effective cost, and ease of fabrication. It is envisioned that the proposed HCC sensing platform and the HCC-FPR sensing technique can be modified for measurements of other parameters in harsh high-temperature environments such as pressure, acoustic wave, vibration, temperature, molten steel level, torque, and twist.

## Supplementary Materials

The following are available online at http://www.mdpi.com/1424-8220/18/5/1304/s1, Video S1: Sensor Video Demonstration.
